# Queene Elizabethes Achademy

**Published:** 1920-03

**Authors:** L. M. Griffiths


The Bristol

Medico-Chirurgical Journal.

*"Scire est nescire, nisi id me
Scire alius sciret."*

MARCH, 1920.

### QUEENE ELIZABETHES ACHADEMY.

(British Museum, *Lansdowne MS.* 98.)

### A SIXTEENTH-CENTURY SCHEME FOR A LONDON UNIVERSITY.

CONTRIBUTED BY

L. M. Griffiths, M.R.C.S. Eng., L.R.C.P. Ed.

Sir Humphrey Gilbert (1539–1583), who was half-brother to Walter Raleigh, laid before the Queen (*c.* 1570) a MS. dealing with a plan for "The erection of an Achademy in London for educacion of her Maiestes Wardes, and others the youth of nobility and gentlemen." Gilbert brings before Elizabeth the desirability of the scheme by saying:—

"ffforasmuch as (moste excellent soveraigne) the moste parte of noble men and gentlemen that happen to be your Maiestes Wardes, the Custody of their bodies beinge of bownty grawnted to some, in rewarde of service or otherwise, not without your honorable Confidence of their good educacion, yet, neverthelesse, most commonly by such to whom they are committed, or by those to whom such Committees have sould them, being eyther of evil religion, or insufficient qualities, are, thorough the defaltes of their guardens, for the moste part brought vp, to no small grief of their frendes, in Idlenes and lascivious pastimes, estranged from all serviceable vertues to their prince and Cowntrey, obscurely drowned in educacion for sparing Charges, of purpose to abase their mindes, leaste, being better qualified, they should disdaine to stowpe to the mariage of such purchasers daughters ; As, also, for that the greatest nomber of younge gentlemen within this Realme are most Conversant abowte London, where your Maiestes Cowrte hath most ordinarie residence ; Yt were good (as I thincke, vnder Your Highnes most gratious Correction,) that, for their better educacions, there should be an Achademy erected in sorte."

F. J. Furnivall—in his edition published by the Early English Text Society in 1869—says: "The plan of the Achademy is in fact one for the establishment of a great London University for the education of youths in the art of political, social, and practical life,—a kind of prototype of the London University so wisely pleaded for of late years by Professor Seeley,[1] which should gather into itself the whole range of modern London teachers and studies. I venture to think that Sir Humphrey's scheme will not detract from his fame for nobleness of spirit, keenness of sight, and directness of aim." (p. xi.)

Furnivall quotes other interesting references to Gilbert from the poet Gascoigne in 1576, *Hakluyt's Voyages*, 1600, vol. iii., p. 145, and Froude's *Short Studies on Great Subjects*, vol. ii., pp. 136–45.

It is of special interest to note the way in which Gilbert dealt with the medical work in the scheme.

There shalbe one Reader of naturall philosophie, who shalbe yearely allowed for the same, .. .. .. .. 40[li.]

There shalbe entertained one Doctor of phisick, who shall one day reade phisick, and another daie Chirurgerie, in the

[1] "A Plea for more Universities" by Seeley appeared in *The London Student* of April, 1868.

Englishe toung, towching all kindes of Vlcers, Sores, Phistiloes, wowndes, &c. Togeather with all kindes of medicines for the same, as well Chimice [Chemical] as otherwise, and shalbe yearely allowed, .. .. .. .. .. .. 100$^{li}$

This Reader shall never alleage [refer to] any medicine, be yt of simples, salues, saltes, balmes, oyles, spirites, tinctures, or otherwise, But that he shall declare the reason philosophicall of euery particuler ingredience for such operacion, And shew his hearers the mechanicall making and working thereof, with all manner of vesselles, furnishes, and other Instrumentes and vtensiles apertaining to the same.

This phisition shall continually practize togeather with the naturall philosophor, by the fire and otherwise, to search and try owt the secreates of nature, as many waies as they possiblie may. And shalbe sworne once euery yeare to deliuer into the Treasorer his office, faire and plaine written in Parchment, without Equiuocacions or Enigmaticall phrases, vnder their handes, all those their proofes and trialles made within the forepassed yeare, Togeather with the true evente of thinges, and all other necessary accidentes growing therby, To thend that their Successors may knowe both the way of their working, and the event thereof, the better to follow the good, and avoyd the evill, which in time must of force bring great thinges to light, yf in Awcomistrie [alchemy] there be any such thinges hidden. ffor whose saffetyes I would wish the Statute of the 5th of Henry the 4th towching multiplicacion to be dispensed at large.

The Phisition shall practize to reade Chirurgerie, becawse, thorough wante of learning therein, we haue verie few good Chirurgions, yf any at all, By reason that Chirurgerie is not now to be learned in any other place then in a Barbors shoppe, And in that shoppe, most dawngerous, especially in tyme of plague, when the ordinarie trimming of men for Clenlynes must be done by those which haue to do with infected personnes.

This Philosophor and phisition shall haue a garden apointed them which they shall furnish and maintaine with all kindes of simples ; and shalbe yearely allowed, besides their Lectures, for their afforesaid extra ordinarie Charge and practizes, 100$^{li.}$

There shalbe one keeper of the Liberarie of the Achademy, whose Charge shalbe to see the bookes there saffely kepte, to Cawse them to be bownd in good sorte, made fast, and orderly set, And shall keepe a Register of all the bookes in the said Liberarie, that he may geve accompte of them when the Master of the Wardes, or the Rector of the Achademy shall apoincte ; and shalbe yearely allowed, .. .. .. .. 26$^{li}$

This Keeper, after every marte [book fair], shall Cawse the bringers of bookes into England to exhibit to him their Registers, before they vtter any to any other person, that he may pervse the same, and take Choyse of such as the Achademie shall wante, and shall make the Master of the Wardes, or the Rector of the Achademy, privy to his Choyse, vpon whose warrante the bookes so provided shalbe payed for. And there shalbe yearely allowed for the buying of bookes for the said Liberary, and other necessary instrumentes, .. .. .. $40^{li.}$

All Printers in England shall for ever be Charged to deliuer into the Liberary of the Achademy, at their owne Charges, one Copy, well bownde, of euery booke, proclamacion, or pamflette, that they shall printe.

There shalbe geven in stocke for the furnishing of a Liberarie and Instrumentes apertaining to the same, Togeather with the buying of horses, as afforesaid,[1] and all other necessary thinges for the first furnishing of this Achademy, .. .. $2000^{li.}$

The whole yearely wages and Charges of this Achademy amownteth vnto .. .. } $2507^{li.}$ $6^{s.}$ $8^{d.}$

The whole yearely Charges for the Commons of the said Readers, officers, and seruantes in this Achademy amownteth vnto } $459^{li.}$ $6^{s.}$ $8^{d.}$

which maketh yearely in all .. .. $2966^{li.}$ $13^{s.}$ $4^{d.}$

Here wanteth levyes for the building or buying of howses for this Achademy.

All the publique Readers of arte and the common lawes shall once within every six yeares set forth some new bookes in printe, according to their severall professions.

Also every one of those which shall publiquely teache any of the languages as afforesaid, shall once every 3 yeares publish in printe some Translation into the English tounge of some good worke, as neare as may be for the advawncing of those thinges which shalbe practized in the said Achademy.

All which bookes shall for ever be entituled as set forth by

[1] The reference to this is an earlier portion of the MS. in these terms : "There shalbe entertained into the said Achademy one good horsman, to teache noble men and gentlemen to ride, make, and handle, a ready horse, exercizing them to runne at Ringe, Tilte, Towrney, and cowrse of the fielde, yf they shalbe armed. And also to skirmish on horsbacke with pistolles, not taking for the learning of any one of them above 10s. by the moneth, he finding them horses for that purpose, and shalbe ready bownd to keepe theare 10 greate horses for the said exercize, beinge yearely allowed therefore, 333li. 6s. 8d. This Rider shall haue allowed vnto him at the first erecting of the Stable, to buy his horses, 266li. 13s. 4d. This Rider, at his first Coming vnto the office, shall enter into bondes with suffitient sureties to leave vnto the Achademie, at his death or departure, the said horses, in asgood estate as he receaved them, or others as good, or the full summe which he was allowed for the buying of them."

the gentlemen of Queene Elizabethes Achademy, wherby all the nations of the worlde shall, once every 6 yeares at the furthest, receaue greate benefitt, to your highnes immortall fame.

At this present, the estate of gentlemen cannot well traine vp their children within this Realme but eyther in Oxford or Cambridge, whereof this ensueth :

ffirst, being theare, they vtterly lose their tymes yf they doe not follow learning onely. ffor there is no other gentlemanlike qualitie to be attained.

Also, by the evill example of suche, those which would aply their studies are drawen to licentiousnes and Idlenes ; and, therefore, yt were every way better that they were in any other place than theare.

O noble prince, that god shall blesse so farre as to be onely meane of bringing this seely [simple], frosen, Island into such everlasting honnour that all the nations of the World shall knowe and say, when the face of an English gentleman appeareth, that he is eyther a Sowldiour, a philosophor, or a gallant Cowrtier ; whereby in glory your Maiesty shall make your sellf second to no prince living.

And whereas the fame of the noblest Conquerors that ever were is onely renewed by history, your maiesty shall not onely haue your share thereof, but also for evermore, once every 3 or 6 yeares at the most, fill the eyes of the world with new and chaunge of matter, wherby all sortes of Studentes shalbe alwaies put in minde of Queene Elizabethes Achademy. And in the mean tyme, the pervsing of the old, and expectacion for the new, shall occupy Continually euery mannes tounge with Queene Elizabethes fame. So that your maiesty, being deade, shall make your sepulchre for ever in the mowthes of the livinge.

To conclude, by erecting this Achademie, there shalbe heareafter, in effecte, no gentleman within this Realme but good for some what, Wheareas now the most parte of them are good for nothinge. And yet therby the Cowrte shall not onely be greatly encreased with gallant gentlemen, but also with men of vertue, wherby your Maiesties and Successors cowrtes shalbe for ever, in steade of a Nurserie of Idlenes, become a most noble Achademy of Chiuallric pollicy and philosophie, to your greate fame. And better it is to haue Renowme among the good sorte, then to be lorde over the whole world. ffor so shall your Maiesty make your self to live among men for ever (whereas all flesh hath but small continuaunce), and therwithall bringe youre selfe into goddes favour, so farre as the benefittes of good workes may prevaile.

Taking into consideration the disparaging references to Oxford and Cambridge, it is not improbable that Gilbert was led to propose his scheme by a desire to find a remedy to correct the deficiencies of these Universities, which were most marked in his time. If that is the case, his estimate is amply confirmed by recent writers on the conditions in the Elizabethan period. Emphasis has been laid on the University " intellectual insignificance " which then existed as the outcome of the Royal Injunctions of 1535 issued by Thomas Cromwell.

Ignoring the legends (common to both these Universities) about their beginnings, we may accept the statement that King Alfred founded, on Grammar School lines, an Academy at Oxford, which almost certainly was the origin of the University, that did not take anything like its present form till some centuries later. We should bear in mind that for a long period this Association of Scholars was under monastic and Papal regulations. This state of things was also similar at Cambridge till Reformation times.

It is possible that the visits of Elizabeth to Oxford and Cambridge had something to do with questions connected with Gilbert's 1570 Scheme. In 1571 Robert Dudley, Chancellor, was responsible for incorporating " the Chancellor, Masters, and Scholars," and for his influence for intensifying the strong position of Puritanism. In the same year Elizabeth granted a Charter for the foundation of Jesus College in Oxford. Subscription to the Act of Supremacy and Uniformity was imposed in her reign, during which Parliament passed an Act conferring economic and domestic advantages on both Universities, foreshadowing some of the Food difficulties of the present day.

Gilbert did not foresee the advantages which were to result from bringing together the students of all the Colleges to discuss questions of far-reaching importance. The "Unions" of Oxford and Cambridge were not founded till the early part of last century, and these combine the objects of Literary Club and Debating Society. London University is now contemplating the formation of such an institution for its students. (See *The Observer*, London, March 21st and 28th, 1920.)

---

